# Bridgewater Treatises

**Published:** 1836-04-01

**Authors:** 


### THE BRIDGEWATER TREATISES.

### Chemistry, Meteorology, and the Function of Digestion, considered with reference to Natural Theology. By *William Prout*, M.D. F.R.S. &c.

There have been serious doubts whether the pious and philanthropic expectations of the late Earl of Bridgewater have been fully realized. It is impossible to admire or to laud too highly the spirit which dictated the appropriation of so large a sum as eight thousand pounds, from the property of a private individual, to the exclusive service of religion. It was a similar spirit that, in the middle ages, induced the superstitious Catholic to render a posthumous atonement for real or imaginary crimes, by founding receptacles for men whom laziness or mistaken piety secluded in the cloister. A spirit, similar in kind, but different in its rationality. We cannot conceive a more striking instance of enlightened devotion, than the dedication of a princely sum to the diffusion, among the careless and the ignorant, of a knowledge of the wisdom and goodness of their God.

But, as we observed, it has been seriously doubted, whether those who superintended the execution of the bequest adopted the best means of giving it effect. We admit the difficulties under which they laboured, and the impossibility (we might almost use that term) of procuring, at a minute's warning, men able and willing to handle, at another's bidding, a deep and complex question in a masterly way. The result has been what was, in some degree, anticipated. A series of bulky volumes have issued from the press, in which the single argument of design has been so diluted and dilated, that it has lost in quality more than it has gained in quantity. Did we wish to convince an atheist of his error, we would refer him to Paley, not to the Bridgewater Treatises. Paley has chosen his ground and managed his case as a logician would. The writers of the treatises have been compelled to employ much mere argument and conjecture, and to urge probabilities as well as proofs. Those who are acquainted with the human mind, know that one sound argument, strong in all its parts, goes farther to convince unwilling reason than many arguments, parts of which are not strong. If Paley proves design by the mechanism of a watch, he also proves design by the mechanism of a muscle. The very simplicity of the reasoning is its force. The man who contravenes, or tries to contravene it, opposes the common experience of mankind; and that is the very thing to be desired. We do not fully agree with Dr. Prout on this point. In the treatise lying before us, that able physician remarks :—

"When a series of wheels or of levers are arranged in a certain order, they *must* move in a certain way, and produce a certain effect which can be foretold exactly. In such a case, we may admire the skill and ingenuity of the Contriver, or perhaps feel astonished at his power, but we scarcely do more, and much of the effect is lost in the apparent necessity of the result, and the consciousness that under the circumstances nothing else could have happened. When the Deity, therefore, operates through the medium of mechanism, he appears almost too obviously to limit his powers within the trammels of necessity; but when he operates through the medium of chemistry, the laws of which are less obvious, and indeed for the most part unknown to us, his operations have much more the character of those of a free agent, and, in many instances also, appear of a higher order, and are more striking and wonderful. Do not, for instance, those extraordinary and mysterious changes constantly going on around us, beneath us, within us, derive no small additional interest from the very circumstance of their not being understood? 11.

This view is correct or not, just as we do or do not consider it in the light and nature of an *argument*. If a thing is to be proved, the plainer and simpler the method of proof, the more efficient it is for its purposes. It may enhance our estimation of the powers of the Deity, to contemplate the incomprehensible wonders that surround us, if we commence the contemplation of those wonders impressed with the belief that there *is* a Deity. But it must be obvious that, in proportion as they are unintelligible, they are less and less adapted to form an ab initio argument in favour of the Deity's existence. First prove that there is a God, by arguments the most simple and logical that can be found—then gradually heap up the mass of cumulative evidence, which, although in logic it may not strengthen the more positive primary data and conclusions, yet, to the common sense of mankind, offers additional reasons for belief.

From what we have seen of the Bridgewater Treatises, we should say that they are rather adapted to confirm faith than to create it. The fact is, that Paley has left the great argument for natural religion almost in a perfect state. If design proves the existence of a God, Paley has proved the existence of design. All that can be done, is to add variety and number to his illustrations, and to charm the reader, who has learnt to recognize the hand of a Maker, with the fresh examples that the progress of science is continually unfolding of His wisdom, and power, and goodness. And this is, in truth, a noble office—the most universal prayer that human intellect could frame as a tribute to the Deity. What can we conceive more worthy of all the beneficence He has displayed, than the picture of his creatures exerting the reason He has granted in exploring the penetralia of Nature, for materials to form the theme of gratitude and adoration to Himself.

We are led to notice the present volume, because it gives us pleasure to second, in our small way, the benevolent intentions of the Earl of Bridgewater, and to disseminate among the members of our profession, too many of whom are accused of levity, if not of actual irreligion, a more extended knowledge of the great natural truths, than the exclusive practice of medicine can afford. A writer in another journal* has broadly charged us with impiety. We all know that there is a set of persons who measure true piety by their own opinions, whose god is the fiery god of bigots, and who, on all occasions are too well disposed to—

> Deal damnation round the land,
> On each *they* judge His foes.

We will not say that the writer to whom we have alluded belongs to that unhappy description of men; but we can only award him charity at the expense of understanding. True friends to religion, and sincerely desirous of promoting its best interests, we cannot see, without sorrow, its injudicious

---

* The Medical Gazette.

advocates exposing it to ridicule and contumely, by their indecent though well-intentioned efforts to introduce its mysteries into all companies and all discussions. They are eternally handling the Ark, as if the divine fabric of Christianity needed this incessant plastering and propping.

We must not forget, that the arguments for natural and revealed religion are essentially distinct. Their proofs are separate, and so should be their consideration. Natural religion is established by the palpable design displayed throughout the scheme of nature. Revealed religion rests on the force of testimony and the accomplishment of prophecy. So strong is that evidence, so complete the fulfilment of those prophetic missions, that the most acute reason and most towering intellects have submissively allowed their force. But gentlemen like Mr. Kirby, anxious, yet not discreet in their anxiety, to offer to revealed religion extraneous support, have unwarily made it rest on evidence and argument which were not intended by the Almighty to prove it, and cannot, from the nature of things, do any thing but prejudice it. When, for instance, Mr. Kirby gravely attempts to fortify revelation by fancies so crude as to be laughable, and hypotheses so groundless as not to admit of sober discussion—when Mr. Kirby gravely does this, we conceive that the best friend of religion is he who checks the gratuitous injury to her cause. Such, at all events, is our conviction, and, though we may be wrong, we will not quietly permit any man, publicly or privately, to impugn intentions, which he arrogantly assumes the power of divining. Those intentions, we are conscious, will bear the strictest scrutiny, not only of others, but, what is more important, of ourselves; and we endeavour, to the best of our ability, to give them effect. But we gladly drop this unpleasant topic.

The work before us is devoted, as the title tells us, to chemistry, meteorology, and the function of digestion. Much in such a treatise must be elementary; and, whether elementary or not, the greater portion must be too exclusively consecrated to chemical and philosophical details to admit of notice in a medical review so avowedly practical as this. All that we can do is, to select some parts of the treatise for analysis or criticism, and lay before our readers such facts or opinions as we think adapted for us and for them. This plan, which is unavoidable, will preclude our offering a connected view of the work, and those who are anxious to become acquainted with *all* that it contains, we must refer to the author himself.

The treatise is divided into three books—the first being occupied with the consideration of chemistry—the second with that of meteorology—and the third with that of the function of digestion. Perhaps the natural connexion between the several subjects is not so strong as might be wished, but each is, *per se*, replete with interest to all who are anxious to possess some knowledge of the wonders and variety of Nature.

Chemistry is treated in the following order. After some remarks on the rank of chemistry as a science, and on its application to the argument of design, Dr. Prout considers seriatim—the mutual operation and the laws of physical agents and of matter—the properties of matter, including a view of solids, liquids, and gases—of electricity, magnetism, and polarity—of heat and light; next we are presented with a sketch of the chemical elementary principles, which lead to the laws of chemical combination, and an outline of the atomic theory. This may convey an idea of the plan adopted by our author, who dedicates 178 pages to this portion of his subject. At the end of several of the chapters is a recapitulation of the leading facts, or of the principles enunciated, and to these recapitulations we confine ourselves.

In the opening of the first chapter is a quotation from Paley, which we recommend to the serious attention of those, who, not content with summoning the Deity to a special intervention for their puny interests, at every instant that their wants or passions induce them arrogantly to suppose that He should come—who, not content we say with this, heap opprobrious epithets on the heads of all who are too humble to suppose that the Omnipotent would for their sakes reverse his eternal laws. The passage in question runs thus.

"God has been pleased to prescribe limits to his own power, and to work his ends within those limits. The general laws of matter have perhaps the nature of these limits; its *inertia*, its reaction, the laws which govern the communication of motion, of light, of heat, of magnetism and electricity, and probably of others yet undiscovered. These are general laws, and when a particular purpose is to be effected, it is not by making them wind and bend and yield to the occasion, (for nature with great steadiness adheres to and supports them,) but it is, as we have seen in the eye, by the interposition of an apparatus corresponding with these laws, and suited to the exigency which results from them, that the purpose is at length attained." 16.

That the Creator of such forces as attraction, electricity, magnetism, and life, *could* alter or suspend them, no man in his senses will deny. The question is not if the Deity *can*, but if he *does*. Setting aside the established miracles of Revelation, we join with Paley, who was no mean reasoner, in thinking it more consistent with what we know of Nature, and with what we may suppose of God, to believe that He does not. But, however that may be, we are sure that it is at once indecent and disgraceful, to assail men for opinions which they hold in common with those who have adorned Religion, Christianity, and the Church. These are the muddy dregs of that ferocious ignorance which persecuted "the starry Galileo."

Perhaps some of our readers may be unconscious of the extreme divisibility of matter; or rather they may not be exactly aware of the calculations which ingenious men have made. As an abstract possibility, it seems equally difficult to suppose matter divisible ad infinitum, and to suppose that divisibility to stop. Such is the constitution of our minds, that although we are compelled to admit that an ultimate molecule there must be, yet we cannot conceive a molecule so small, as not to be made up of molecules smaller. The following, however, are the calculations on the subject. Dr. Thomson has shewn that an ultimate molecule of lead cannot weigh more than the $\frac{1}{310\cdot000\cdot000\cdot0000}$th, nor an ultimate molecule of sulphur more than $\frac{1}{2\cdot015\cdot000\cdot000\cdot000}$th of a grain, and probably a great deal less; and that the *size* of the molecule of lead cannot surpass, and is probably much less than the $\frac{1}{888\cdot492\cdot000\cdot000\cdot000}$th of a cubic inch!* The vegetable kingdom presents us with innumerable instances, not only of the extraordinary divisibility of matter, but of its activity, in the almost incredibly rapid development of cellular structure in certain plants. Thus the *Bovista* gi-

* System of Organic Chemistry, I. 7.

*ganteum* (a species of fungus), has been known to acquire the size of a gourd in one night. Now supposing, with Professor Lindley, that the cellules of this plant are not less than the $\frac{1}{200}$th of an inch in diameter, a plant of the above size will contain no less than 47·000·000·000 cellules; so that, supposing it to have grown in the course of twelve hours, its cellules must have been developed at the rate of nearly 4·000·000·000 per hour, or of more than sixty-six millions in a minute !* and when we consider that every one of these cellules must be composed of innumerable molecules, each one of which is again composed of others, we are perfectly overwhelmed with the minuteness and number of the parts employed in this single production of nature. In the animal kingdom, monads have been discovered so small, that a million of them do not exceed in size one grain of sand. Yet each particular monad displaying, as it does, sensitive, loco-motive life, must have its special organization. How wonderful the Deity—now contriving the muscles of a monad, now bidding a planet to swing in space !

Dr. Prout indulges in a pleasing theory on the cause of the chemical and cohesive attractions of the molecules of matter. We have not time to go into the theory itself, which, whatever its merits, is at present but a theory. The following recapitulation of the author embraces his principal conclusions, though not the data nor the reasoning that led to them.

"1. In the first place," says Dr. Prout, "we attempted to show that the forces which determine molecular union can scarcely be those of mere gravitation, in their ordinary forms at least; but that some other modification of force is necessary to account for the phenomena.

2. By assuming the molecules of bodies to be virtually spheroidal, and endowed with two kinds of polarizing forces, the one operating *axiallay*, and the other *equitorially*, we attempted to show how the phenomena of simple crystallization might be explained; and we corroborated our argument by demonstrating that the electric and magnetic forces are actually related to each other, precisely as we assumed the energies of our molecules to be. Hence we ventured to draw the conclusion, that electricity and magnetism, if not identical with, at least represent, or are analogous to those forces, the existence of which among ponderable bodies we assumed as necessary to account for the phenomena of crystallization. Further, we attempted to render it probable, that the molecules of the imponderable principles, heat and light, possess polarities precisely analogous to those of ponderable bodies, and that many of their peculiar phenomena depend upon these polarities.

3. In attempting to account for the different forms assumed by bodies, we supposed that in the *solid* form, the molecules are so arranged as to attract each other according to certain laws; that in the *liquid* form, they are so arranged as neither to attract nor repel each other; and that in the *gaseous* form, the arrangement of the energies of the molecules is such as to render them mutually repulsive. Further, by assuming that those molecules which possess the property of attracting each other in the solid form in preference to others, retain a similar relation in the gaseous form, and repel each other in preference to others, we attempted to account for many of the well-known phenomena of gaseous bodies.

4. Lastly, we attempted to show that the phenomena of *radiation* among the molecules of imponderable bodies, are precisely analogous to the phenomena of

* Introduction to Botany, page 7.

diffusion and mixture among the molecules of ponderable bodies when in the liquid and gaseous states; and that consequently the same laws are strictly applicable to both."

The extract is long, but as it gives a resumé of the opinions of our author, it may not be uninteresting to those who are not in possession of the book.

The argument drawn from the molecular constitution of bodies, is not we think so forcible as some very able men have deemed it. The argument mainly rests on this:—that the very divisibility and molecular constitution of matter, are proofs that its present form has not been eternal, but the result of intelligence. Observe that the argument is not that this or that particular arrangement shews design, but simply that divisibility and molecular composition shew design. All hinges on the non-eternity of molecules. Let us hear what Dr. Prout advances:—

"Although we can form no idea of what matter would be without its molecular properties, there is yet nothing in these properties which can induce us to believe that they are *necessary* to the mere *existence* of matter. On the contrary, we have seen that matter possesses qualities (those of gravitation) of a more primordial kind, to which its molecular properties are apparently secondary or subordinate. But if these subordinate properties be not necessary to the existence of matter, matter might *possibly* at some time have existed without them. Now this very possibility is incompatible with eternal existence; for eternal (passive) existence necessarily involves incapability of change. Hence the molecular constitution of matter, even in this point of view, must be supposed to have had a beginning; and when we consider the leading and characteristic property of matter in the molecular state, viz. *the endless repetition of exactly similar parts,* the difficulty of arriving at any other conclusion is exceedingly increased." 87.

We confess we do not feel exactly as Dr. Prout appears to do with respect to the constitution of matter. If he can *conceive* matter to have existed without a molecular composition, and without some at least of the molecular properties, we own that we cannot do so. He instances the pre-existence of the property of gravitation; but gravitation seems to us to presuppose the presence of molecules. What is gravitation but the attraction of one molecule towards another? We cannot imagine it to be any thing else. If then one general molecular property be conceived to be eternal, another general molecular property may as easily be conceived to be eternal also. Analogy will not serve our author, for no analogy with which we are acquainted is in favour of the *necessary* pre-existence of only one homogeneous matter, without molecules or molecular properties.

Arguments drawn from metaphysical possibilities or impossibilities, are seldom of much value. These are the ultimate facts with which our reason struggles to no purpose.

In vain we dive
In our lone wanderings to the caves of death;
Searching its cause in its effect, and draw
From withered bones, and skulls, and heaped up dust,
Conclusions most forbidden.

If we descend from the speculative regions of the molecular constitution of matter, to the grosser and more tangible compositions, that our senses, our instruments, and our analyses appreciate, we see no longer the misty chain of possibilities, but the certain evidences of wisdom and design.

Those evidences bring wholesome conviction in their train, while the subtle disquisitions of philosophy or fancy—disquisitions as various as the individuals who indulge in them, pass over the mind like a fantastic dream, and leave no substantial or permanent image behind. We would repeat what we have said before—that, for argument and proof, the more simple and obvious instances are best—but, as subsidiary to proof, as means of exciting our admiration, our awe, and our gratitude towards the Supreme Creator, the great operations of Nature, which rather logically infer design than prove it, should be resorted to by the theologian.

We must make one stride from the commencement of the portion of the work which treats of chemistry, to its conclusion. In the recapitulation which we find here, the author points out the adaptation of one thing to another, which we observe in our survey of the Universe. He dwells, for example, on the relation of the water to the temperature of the earth. Supposing that temperature to have been much lower, the water must have been frozen—supposing it to have been much higher, the water must have been converted into vapour. Unfortunately, however, this is, like many of the general facts and laws of Nature, not of much service as a mere argument. The sceptic, pointing to the telescope, observes—in other planets, you see that there must be other temperatures than that which exists in connexion with the earth, and, in them, matter takes its form and its consistence in accordance with it. But, when we prove design in small things, and we perceive adaptation in great, that mind is strangely constituted which, whilst it cannot refuse the palpable existence of contrivance in the valves of the aorta, contends that there is none in the arrangements of that world, on the surface of which the beings with aortæ form scarcely perceptible atoms. In fact, there are but two human beings who *can* be atheists—a downright idiot, with no reason at all—or a downright metaphysician, who refines his reason away.* With this dogmatical, though, we think, not very incorrect conclusion, we take our leave of the subject of chemistry. The whole of the last chapter upon it, in the treatise, is creditable to the head and heart of the author.

The second book, to which we now proceed, treats of meteorology, and embraces a general sketch of the constitution of the globe, and the distribution and mutual influence of the agents and elements of chemistry in the economy of nature. We cannot, of course, pursue our author in this discursive range over sea and land, but shall confine ourselves to noticing such facts respecting climate as professionally interest us. The consideration of this is taken up in the second section of the third chapter.

After stating the common geographical division of the surface of the globe into torrid, temperate, and frigid zones, Dr. Prout considers, seriatim, the temperature of each. The poles and the equatorial regions represent, of course, the extremes.

We are yet deficient in exact information on the mean temperature of the polar regions. It appears that, if we attempt to calculate the temperature of the North Pole by the temperature of the old world, and by that of the new, we obtain very different results; the temperature of the old world in-

---

* Kant was an atheist.—Eds.

dicating the temperature of the pole to be about 10°; while the temperature of the new world indicates it to be considerably below Zero. Hence it has been inferred, that there are two points or poles of greatest cold situated in about the latitude of 80° north, and in longitudes 95° east, and 100° west; and consequently that the geographical pole of the globe is not the coldest point of the Arctic hemisphere. Whether this deduction be well founded or not, must be decided by future observation. At present, the actual temperature of the Polar regions cannot be considered as determined.

The lowest *authentic* observations of temperature that we possess, are those by Captain Parry at Melville Island. Here the thermometer in the ship was often observed as low as 50°; and, at a distance from the ship, even as low as 55° under Zero. The greatest degree of cold produced artificially has been 91° under Zero.

"The mean annual temperature of the equatorial regions (says our author), like that of the polar region, is a meteorological problem of considerable interest. Humboldt, from a very extensive generalization, fixed the mean equatorial temperature at 81½°; and the same temperature has been adopted by others. Attempts, however, have been recently made to shew that this temperature is 3° or 4° below the truth; but Humboldt in reply still maintains his former opinion. Since at the equator, only about one-sixth of the whole circumference of the globe is dry land, the general equatorial temperature, as actually found to exist, is perhaps lower than upon theoretical principles it ought to be; and certainly much below what it ought to be, as deduced from observations made on the continent in the neighbourhood of the equator. Thus the mean temperature of Pondicherry, in latitude 11° 55′ north is at least 85°; and if from this temperature that of the equator were deduced according to the common principles, the deduction would of course be much above the truth. The fact is, as in the case of the Polar regions, we do not possess the requisite data for determining the equatorial temperature in a perfectly satisfactory manner." 211.

Very high temperatures have occasionally been observed in the equatorial regions. At Benares, the thermometer has been recorded as standing at 110°, 113°, and even at 118°. At Sierra Leone, it has been noticed to shew upon the ground a temperature of 138°. Humboldt gives many instances of the temperature of the surface of the earth, amounting to 118°, 120°, and 129°; and on one occasion he found the temperature of a loose and coarse granitic sand to amount to upwards of 140°, the thermometer in the sun at the time only indicating a temperature of about 97°.

The temperature of the regions of the globe intermediate between the polar and the equatorial, presents a subject of equal interest and importance to the philosopher and the physician. The great geographical division of "temperate zone" includes so many places of very various temperatures, in the same parallels of latitude or of longitude, that, for all precise or useful purposes, it is totally effete, and superseded by what is termed the *Isothermal* arrangement. According to this, all the places upon the globe, having *the same annual mean temperature* are classed together; and lines drawn upon a map through such a series of places, have been termed *Isothermal lines*, or *lines of equal temperature*. Now these Isothermal lines are not regular, nor parallel to the parallels of latitude. The new continent is colder in the same parallel of latitude than the old, and, consequently, if we take the northern hemisphere, an *Isothermal line* is nearer the pole in the old world than in the new; in other words, places in the *same* parallel of latitude are warmer in the former than they are in the latter. This is a valuable fact.

Humboldt has divided the northern hemisphere into six Isothermal bands or zones, viz.

| | |
|---|---|
| 1. The zone of mean annual temperature ranging | from 32° to 41°. |
| 2. . . . . . | from 41° to 50°. |
| 3. . . . . . | from 50° to 59°. |
| 4. . . . . . | from 59° to 68°. |
| 5. . . . . . | from 68° to 77°. |
| 6. . . . . . | from 77° upwards. |

As an instance of the differences of temperature in different places in the same parallel of latitude, we may state that the Isothermal line of 32° ranges through a space of 14° or 15° of latitude, and that its western extremity, in the central parts of America, is 5° or 6° nearer the equator than its eastern extremity in Siberia, a striking illustration of the cold of the new continent. In proportion as they approach the equator, the Isothermal lines become less and less curved, so that the line of 77° differs but little from a straight line coincident with the tropic of cancer.

Isothermal lines are drawn from the mean temperatures of the whole year; but it must be useful to ascertain the places in which the mean temperature of portions of the year are similar. Lines drawn through places with the same Summer and the same Winter temperatures are denominated Isotheral and Isocheimal lines. The following observations of Humboldt, on the actual distribution of temperature in the Northern Hemisphere, are usefully quoted by Dr. Prout. We cannot do better than extract them.

"'The whole of Europe (says Humboldt) compared with the eastern parts of America and Asia, has an insular climate; and upon the same Isothermal line the summers become warmer, and the winters colder, as we advance from the meridian of Mont Blanc towards the east or the west. Europe may be considered as the western prolongation of the old continent; and the western parts of all continents are not only warmer at equal latitudes than the eastern parts; but even in the zones of equal annual temperature, the winters are more rigorous, and the summers hotter on the eastern coasts than on the western coasts of the two continents. The northern part of China, like the Atlantic region of the United States, exhibits seasons strongly contrasted; while the coasts of New California and the embouchure of the Columbia have winters and summers almost equally temperate. The meteorological constitution of those countries in the north-west resembles that of Europe as far as 50° or 52° of latitude. In comparing the two systems of climates, the concave and the convex summits of the same Isothermal lines, we find at New York the summer of Rome and the winter of Copenhagen; at Quebec, the summer of Paris and the winter of St. Petersburg. At Pekin, also, where the mean temperature of the year is that of the coasts of Brittany, the scorching heats of summer are greater than at Cairo, and the winters are as rigorous as at Upsal. So also the same summer temperature prevails at Moscow, in the centre of Russia, as towards the mouths of the Loire, notwithstanding a difference of 11° of latitude; a fact that strikingly illustrates the effects of the earth's radiation on a vast continent deprived of mountains. This analogy between the eastern coasts of Asia and America sufficiently proves,' continues Humboldt, 'that the inequalities of the seasons depend on the prolongation and enlargement of continents towards the pole; on the size of seas in relation to their coasts; and on the frequency of the north-west winds, and not on the proximity of some plateau or elevation of the adjacent lands. The great table lands of Asia do not stretch beyond 52° of latitude ; and in the interior of the new continent, all the immense basin bounded by the Alleghany range, and the rocky mountains, is not more than from 656 to 920 feet above the level of the ocean.' " 217.

These statements are more explicitly detailed in an appendix to the present work, where Dr. Prout has inserted a table of temperatures, taken from the article Meteorology, in the Encyclopædia Metropolitana. We would almost wish to transfer the table to our pages, containing, as it does, information always useful to the medical practitioner. But we shall limit ourselves to selecting a few places, and stating their temperatures at various seasons.

At St. Petersburg, which is in the Isothermal zone of from 32° to 41°, and the latitude and longitude of which are 59° 56′ north, and 30° 19′ east, the mean annual temperature is 38·84. The mean temperature of the Winter is 17·06—of the Spring, 38·12—of the Summer, 62·06—of the Autumn, 38·66—of the *warmest* month, 65·66—and of the *coldest* month, 8·60. Thus, the range between the *mean* temperatures of the warmest and the coldest months, at St. Petersburg, is 57·6.

At Moscow, the mean temperature of the year is 40·10—of the Winter, 10·78—of the Summer, 67·10—of the warmest month, 70·52—and of the coldest month, 6·08. The range at Moscow is extremely great—so much as 64·44, between the extreme *mean* temperatures. The most equable temperature, in this Isothermal zone, would seem to be at the North Cape, where the mean annual temperature is 32·00—the mean temperature of the warmest month, 46·58—of the coldest month, 22·10—the variation only 24·48.

In the next Isothermal zone, from 41° to 50°, we find, amongst other places, Quebec, Copenhagen, Dublin.

At Quebec, the mean temperature of the year is 41·74—of the Summer, 68·00—of the Winter, 14·18—of the warmest month, 73·40—of the coldest, 13·81. The range at Quebec is very nearly 60°.

At Copenhagen, the mean annual temperature is 45·68—the mean temperature of Winter, 30·74—of Summer, 62·60—of the hottest month, 65·66—of the coldest month, 27·14—the range, 38·12.

At Dublin, the mean annual temperature is 49·10—of Winter, 39·20—of Summer, 59·54—of the hottest month, 61·16—of the coldest, 35·42.

At the Malouine Islands, in the latitude of 51° 25′, the temperature is more equable than at any other place in this zone. Their mean annual temperature is 46·94—that of Winter, 39·56—that of Summer, 53·06—of the warmest month, 55·76—of the coldest, 37·40, the range being only 18·36.

In the Isothermal zone, from 50° to 59°, we will take Paris, London, and New York.

The mean temperature of Paris for the year is 51·08—of the Winter, 38·66—of the Summer, 64·58—of the hottest month, 65·30—of the coldest, 36·14—the variation, 29·16.

In London, the mean annual temperature is 50·36—of Winter, 39·56—of Summer, 63·14—of the hottest month, 64·40—of the coldest month, 37·76—the variation, 27·64. Thus, London is cooler than Paris in the Summer, warmer in the Winter, and exhibits a minor range of variations in temperature.

At New York, the mean annual temperature is 53·78—of Winter 29·84, (10 degrees below that of London,) of Summer 78·16, (16 degrees above that of London,)—of the hottest month, 80·78—of the coldest, 25·34, the range being 55·44.

The next Isothermal zone, from 59° to 68°, presents, amongst other places, Montpellier and Rome.

At Montpellier the mean temperature of the year is 59·36—of the Winter, 44·06—of the Summer, 75·74—of the warmest month, 78·08—of the coldest month, 42·08—the variation 36.

At Rome the mean temperature of the year is 60·44—of the Winter, 45·86—of the Summer, 75·20—of the warmest month, 77·00—of the coldest month, 42·26.

In the Isothermal zones from 68° to 77°, we have only Funchal and Algiers reported.

At Funchal, the mean temperature of the year is 68·54—of the Winter, 64·40—of the Summer, 72·50—of the warmest month, 75·56—of the coldest month, 64·04—the variation being only 11·52.

We will not extract the mean temperature of any places in the Isothermal zones above 77 degrees. We may simply observe, that the variations between the hottest and the coldest months are comparatively slight.

It is evident that, as a means of estimating the salubrity of places, *mean* temperatures are defective. The frequency and suddenness of changes of temperature must also be taken into the account. We may readily imagine that a place may have precisely the same mean temperatures as London, and yet those temperatures may be much more steady. The mischief of the English climate is its variability. As far as range of temperature goes, perhaps England is more favoured than any other country; but then the advantage is in a great measure lost by the capricious variations from wet to dry, and from hot to cold, that occur with the most astonishing rapidity.

Having considered the temperature of the globe, Dr. Prout proceeds to investigate the nature of climate. He admits the difficulty of determining the nature and force of the varied agencies that constitute in combination what we denominate climate, but he thinks that its numerous constituents may be naturally divided into two great sections, viz. *those of a* PRIMARY *kind depending upon the globular figure of the earth; upon its motion in its orbit, and upon its axis: and those of a* SECONDARY, *or subsidiary kind, more immediately connected with the globe itself, and depending upon the nature of its surface, as composed of land or water;* or, *as connected with its atmosphere.*

Dr. Prout accordingly considers in succession the temperature of the earth, as dependent on its globular form, and on its annual and diurnal motions, and the circumstances connected with the surface of the globe and with its atmosphere, that are capable of exerting an influence on climate. We cannot follow our author in the details, embracing as they do phenomena that belong to the science of natural philosophy. The most that we can do is to pick out here and there what may possibly amuse or instruct our readers.

They must be aware of the great discoveries that have been made by Mr. Faraday on the connexion between electricity and magnetism. Now Dr. Prout attempts to account for the magnetic directive property of the earth, (that is, for the property which gives its polarity to the needle,) by its diurnal revolution on its axis from West to East. He is obliged to assume the existence of electric currents circulating within the earth from East to West in planes parallel to the magnetic equator. This is the passage in question.

"We have already alluded to the opinion that heat occasionally passes into the electric and magnetic energies; an opinion which some consider to derive much probability from the phenomena of what has been termed *thermo-electricity;* that is to say, electricity (and magnetism) developed by the unequal distribution of heat through bodies. Now, whether the phenomena of thermo-electricity actually depend on the decomposition of heat, latent or sensible, or upon any other cause, is of little importance; the phenomena themselves are well established, and they seem to account, in the most satisfactory manner, for the general distribution of electricity and magnetism over the earth. The explanation is this: the earth during its diurnal motion on its axis from west to east, has its surface successively exposed to the solar rays in an opposite direction, or from east to west. The surface of the earth, therefore, particularly between the tropics, will be heated and cooled in succession, from east to west, and currents of electricity, on thermo-electric principles, will at the same time be established in the same direction: now these currents once established from east to west, will, of course, give occasion to the magnetism of the earth from north to south. Hence the magnetic directive power of the earth, in a direction nearly parallel with its axis, is derived from the thermo-electric currents, induced in its equatorial regions by the unequal distribution of heat there present, and depending principally on its diurnal motion." 233.

We do not profess ourselves competent to argue the probabilities of this theory. But we are quite certain that it is not proved, and that it is premature founding an argument upon it. Yet it seems impossible to conceive what *may* result from the researches now going on in electro-magnetism. We are possibly on the threshold only of our acquaintance with the phenomena of Nature.

Dr. Prout points out the benevolence displayed in the arrangement of colours, or rather in giving animals the perception of them. It is a great source of pleasure. Why should it have been, if not for the benignant purpose of occasioning gratification?

Perhaps it may not have occurred to many what consequences would have ensued had snow been dark, or had whiteness prevailed in the equatorial regions. In those regions, the solar heat and light, instead of being absorbed, would have been reflected, and the glare of light and accumulation of heat in the atmosphere contiguous to the surface, would have been incompatible with the existence of the present race of animals. The surface of the earth itself would have been heated slowly, but overheated at last, in consequence of its low radiating powers. Such would have been the case near the Equator, whilst at the Poles, all the little heat and light would have been absorbed. There must have reigned perpetual darkness, and the sudden melting of the snow on the least exposure to heat and light must have occasioned frequent inundations destructive to the inhabitants of other regions.

Dr. Prout devotes some pages to the propagation of heat below the earth's surface by conduction, and to the propagation of heat and light below the surface of the water. Perhaps the notice of a few facts connected with these subjects may not be disagreeable or uninstructive.

Though the surface of the earth, to the depth of some inches, participates in the fluctuations of the temperature of the atmosphere, yet at a certain distance from the superficies, a determinate stratum is found where the temperature is uniform, or nearly so, throughout the year. Dr. Prout remarks, that there is reason to believe that the temperature of this invariable stratum nearly coincides with the mean annual temperature of the place, and that its depth below the surface in different latitudes ranges between forty and eighty feet. It is chiefly on this circumstance that the uniformity of the temperature of cellars and caves depends. As an instance of the uniformity of temperature in such places, it may be mentioned, that a thermometer placed in the caves under the observatory in Paris, at a depth of about eighty-five feet below the surface, has, during fifty years, scarcely varied more than a quarter of a degree from 11·82 of the centigrade scale; equal very nearly to 53¼ of Fahrenheit. The mean annual temperature of Paris is 51·08.

Dr. Prout gives the results of a few experiments that have been made to determine the variation of the temperature throughout the year, at different depths from the surface down to the invariable stratum.

"In the month of August the temperature of the earth goes on decreasing in nearly a uniform manner, from a little below the surface to the stratum of invariable temperature. In the month of September the temperature is nearly uniform to fifteen or twenty feet below the surface; beyond which depth the temperature decreases a little and slowly to the stratum of invariable temperature. During the months of October and November the temperature increases from the surface to the depth of fifteen or twenty feet; and below this point it remains nearly uniform to the invariable stratum. During December, January, and February, the temperature, being at its minimum upon the surface, increases in a manner nearly uniform, downwards to the invariable stratum. During March and April there is a rapid decrease of temperature to the depth of one or two feet; below this depth the temperature decreases less rapidly; and still lower, the temperature increases a little. During the months of May, June and July, the temperature being at its maximum, at the surface, decreases downwards, but less rapidly and to a greater depth; it then begins to increase a little till it attains the temperature of the invariable stratum. The rapidity and degree, however, with which these changes take place, as well as the changes themselves, appear to fluctuate very considerably, not only in different places under the same Isothermal line, but in the same place in different seasons." 245.

The lateral propagation of heat through the solid parts of the earth is slight.

The propagation of heat and light through water must not long detain us. We shall merely mention one calculation with respect to light, and one fact with reference to heat. It has been calculated, then, that not a tenth part of the incident light can advance five fathoms downwards in the most translucent water; that even of vertical rays, one half is lost in the first seventeen feet, and that they become reduced to one-fourth by traversing thirty-four feet, which correspond to the mass of an atmosphere. It thus follows, that only the hundred-thousandth part of the vertical rays can penetrate below forty-seven fathoms, which is scarcely equal to the glimmer of twilight; and that the depths of the ocean must be always in perpetual darkness. The fact to which we adverted illustrates well the uniformity of temperature at the bottom of deep lakes or inland seas. It has been found that the temperature of the water, at the bottoms of many of the lakes in Switzerland, often varies no more than 3° or 4°, while that of the surface exhibits a range of 20° or 30°.

We are interested professionally in being acquainted with the amount of differences of temperature, occasioned by the influence of land and water.

Far from the land, the diurnal changes of temperature of the air, near the surface of the sea, diminish. In the equatorial regions, the greatest difference between the temperature of the day and that of the night at sea, is said to amount to 3° or 4° only; while upon land the difference often amounts to 9° or 10°. In temperate regions, and particularly in latitudes extending from 25° to 50°, the difference between the maximum and the minimum diurnal range of the thermometer at sea is still very trifling, amounting only to 4° or 6°; while upon the continents, as for example at Paris, the range often amounts to 20° or 30°. Thus, insular climates are more equable than continental.

The minimum temperature is about sunrise—the maximum, at sea, about noon—on land, two or three hours after noon. It appears, from accurate and repeated observations, that even between the tropics, the temperature of the surface of the sea is higher than that of the incumbent atmosphere. Between the latitudes of 25 and 50 degrees, the air is rarely warmer than the surface of the sea; and in the Polar regions it is very unusual to find the air as warm as the sea; it is in fact almost always colder, and generally very much colder.

Dr. Prout occupies a considerable space in the consideration of the constitution and the laws that regulate the atmosphere. The details are far too exclusively philosophical for the pages of a practical medical journal. We cannot attempt to pursue the subject, and will merely refer to such points, here and there, as are connected with medical inquiries.

One such point is the quantity of rain that falls. There is much about both wind and rain to puzzle the *feelosophers*, as Mr. Cobbett called them. The disturbing circumstances are so numerous, as to interfere materially with à priori calculations. But one general fact appears to be made out, that the average quantity of rain that falls diminishes from the Equator to the Poles. Dr. Prout gives a table that illustrates, indeed proves this statement. We need not extract the whole table, for the following instances, taken from it, are sufficient.

At St. Petersburg, the annual fall of rain is 16 to 17 inches—at London, 20 to 25 inches—at Rome, 39 inches—at Vera Cruz, 63 inches—at Logane, St. Domingo, 150 inches.

In this country, Dr. Thomson has estimated that, taking the whole of Great Britain together, the mean fall of rain amounts in the course of a year to 36 inches, the dew being included (which is considered to amount to about four inches); and that the quantity of water evaporated is about 32 inches. The excess of four inches precipitated is supposed to feed the springs and rivers, while the same four inches must be evaporated from our surrounding seas. Yet all these calculations must be taken cum grano salis.

Dew, hoar frost, mists and fogs, snow and hail, appear upon our author's pages. We will not condense on our's all this moisture. Our readers might apply to us the words of Horace's famous ode—

Jam satis terris nivis atque diræ

Grandinis.

But, before we quit rain altogether, we may observe that more rain usually falls near the sea than in the sea—that in mountainous regions, more rain falls than on plains—that the rain chiefly falls at the foot of the mountain, and not upon its top—and, finally, that although less rain falls as we recede from the equator, the number of rainy days increases.

We may pass over the electricity of the atmosphere, and the decomposition of light effected in it; but we may, for an instant, pause to contemplate those peculiar atmospheric states occasioned by the presence of foreign bodies.

The air has been occasionally filled with minute vegetable matters, as lichens and other cryptogamous plants diffused in myriads through it, and sometimes colouring it red. This has given rise to the fabulous stories of *showers of blood.* Of meteoric stones we need say nothing, as Dr. Prout does not add to the stock of general knowledge, or rather ignorance, respecting their origin and nature. But sometimes a peculiar state of air has been observed, exerting a marked influence on the human constitution. In the years 1782 and 1783, a remarkable haze extended over the whole of Europe. This haze was of a pale blue colour. It was thickest at noon, when the sun appeared through it of a red colour. Rain did not in the least degree affect it. This haze is said to have possessed drying properties, and to have occasionally yielded a strong and peculiar odour. It is also said to have deposited in some places a viscid liquid, of an acrid taste, and of an unpleasant smell. About the same time, there were, in Calabria and in Iceland, terrible earthquakes, accompanied by volcanic eruptions. Simultaneously with this atmospheric condition, various epidemic diseases prevailed, particularly an epidemic catarrh, affecting not only man, but animals. Dr. Prout supposes it not improbable, that a minute quantity of some active agent, ejected by a volcanic eruption, and diffused throughout the air, may give, or may have given, rise to epidemic maladies. The following statement is curious, and we subjoin it in our author's words. It deserves, at all events, to be added to the circumstances attendant on the appearance or spreading of cholera, whether it was connected with it or not.

"As an instance of the presence of such bodies in the atmosphere, we may mention a very remarkable observation which occurred to the writer of this treatise during the late prevalence of epidemic cholera. He had for some years been occupied in investigations regarding the atmosphere; and for more than six weeks previously to the appearance of cholera in London, had almost every day been engaged in endeavouring to determine, with the utmost possible accuracy, the weight of a given quantity of air, under precisely the same circumstances of temperature and of pressure. On a particular day, the 9th of February, 1832, the weight of the air suddenly appeared to rise above the usual standard. As the rise was at the time supposed to be the result of some accidental error, or of some derangement in the apparatus employed; in order to discover its cause, the succeeding observations were made with the most rigid scrutiny. But no error or derangement whatever could be detected. On the days immediately following, the weight of the air still continued above the standard; though not quite so high as on the 9th of February, when the change was first noticed. The air retained its augmented weight during the whole time these experiments were carried on, namely, about six weeks longer. The increase of the weight of the air observed in these experiments was small; but still decided and real. The method of conducting the experiments was such as not to allow of an error, at least to an amount so great as the additional weight, without the cause of that error having become apparent. There seems, therefore, to be only one mode of rationally explaining this increased weight of the air at London in February, 1832; which is, by admitting the diffusion of some gaseous body through the air of this city, considerably heavier than the air it displaced. About the 9th of February, the wind in London, which had previously been west, veered round to the east, and remained pretty steadily in that quarter till the end of the month. Now, precisely on the change of the wind, the first cases of epidemic cholera were reported in London; and from that time the disease continued to spread." 352.

Dr. Prout does not affirm that the cholera was actually occasioned by this peculiar state of atmosphere. But he seems to suspect that the two were, in some mysterious way, connected.

After noticing malaria, our author sums up his consideration of climate with a glance at the general arguments it furnishes in favour of Divine contrivance. We need not dwell on them, for those who have resisted the evidence adduced already, and so often, would seek, in metaphysical contradictions, a refuge against the testimony of even an angelic missionary. Gabriel himself could neither confute nor convince them. Let us leave the wretched atheist to live a practical solecism in nature—denying the design without which himself could never have blasphemed—refusing to believe in benevolence which mercifully permits him to escape the instant punishment of his impious mockery—and exerting the very faculties that raise him from the brute, to debase himself and his kind to its level. Let us leave this wretched creature to the comforts of his false philosophy, if philosophy that can be considered which opposes to common sense, and reason, and experience, a buckler of obdurate disbelief. A sceptic the atheist is not. He is a bigot, the most hardened and blind of any—who creates the power that grovelling he adores—and, substituting Nature for Nature's God, would transfer intelligence from the Divinity to the gases and the atoms of which his own putrescent carcase is composed. Poor fool! alike too little and too weak for the vengeance of his Maker, whom he libels, and of human kind that he disgraces. Poor fool! let us hope that the insignificance which escapes the thunderbolt now, may experience the same benignant forbearance hereafter.

The distribution of animals and plants on the surface of the globe is the last topic that occupies our author. We do not think it necessary to pursue him through his interesting but trite details, and we shall close the present article here. In our succeeding Number, we shall devote some pages to the concluding portion of the volume, that treats of the function of digestion. Yet we cannot part, though it be but for a season, from our able author, without recommending such of our readers as can afford the requisite time and means, to peruse the volume before us. It is not adapted for a systematic analysis in a journal such as this; but it *is* adapted to convey to the mind of the professional man, as of the general reader, much pleasing and some useful information. We now take our leave of Dr. Prout for the present.

